# Popcorn Effect–inspired Self‐propagating Formation of High‐conductivity Cement Composite for Multifunctional Applications

**DOI:** 10.1002/advs.202411290

**Published:** 2024-12-04

**Authors:** Haiping Wu, Jiaqi Huang, Zhengyao Qu, Xueling Zheng, Sirui Tan, Wei Du, Guanming Cai, Zhong Zhao, Jing Wu, Daiqi Li

**Affiliations:** ^1^ State Key Laboratory of New Textile Materials and Advanced Processing Technologies Wuhan Textile University Wuhan 430200 P. R. China; ^2^ State Key Laboratory of Silicate Materials for Architectures Wuhan University of Technology Wuhan 430200 P. R. China

**Keywords:** bioinspiration, conductive networks, expandable graphite, portland cement, thermal expansion

## Abstract

The surge in modern civil technologies demands a transformation in cement composites to surpass traditional roles and integrate smart functionalities. In this regard, enhancing the electrical conductivity of cement composites is a critical challenge. This study introduces a novel strategy for the self‐propagating formation of expandable graphite‐based high‐conductivity cement composites through a simple thermal treatment with 3 wt.% expandable graphite and 1 wt.%carbon fiber as conductive fillers. Inspired by the popcorn effect, this method leverages the rapid expansion of graphite at high temperatures, promoting contact between conductive fillers and forming new conductive networks. The obtained composites demonstrate a remarkable reduction of 60% in electrical resistance after heat treatment compared to the electrical resistance of standard cement composites, and the enhancing mechanisms is explored. The conductive properties endow the material with excellent electrothermal (>100 °C at 10 V), electrothermochromic (response time of 2 s), and electromagnetic interference shielding (42 dB at 12.4 GHz) performance. This innovative approach provides vast opportunities for developing smart infrastructure with enhanced electrical properties, regarded as a promising candidate for promoting next‐generation buildings and infrastructures.

## Graphical abstract

This study introduces a novel popcorn‐inspired strategy for self‐propagating expandable graphite‐based conductive cement composites. Through thermal treatment, the graphite expands and forms new conductive networks, significantly reducing electrical resistance by 60%. This enhancement broadens its application scope in electrical applications, including joule heating, electrothermochromic applications, and EMI shielding.

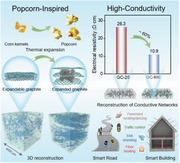

## 1 Introduction

Cement‐based materials form the backbone of the construction industry, with a staggering annual global consumption surpassing 40 billion tons.^[^ ^1^, ^2^, ^3^, ^4^ ^]^ The advent of modern civil technologies has necessitated a paradigm shift in the functionalities of these materials, extending beyond mere structural support to roles.^[^ ^5^, ^6^, ^7^, ^8^, ^9^ ^]^ Electricity is crucial for human civilization, and enhancing the electrical conductivity of cement and concrete is a critical challenge.^[^ ^10^, ^11^ ^]^ At present, considerable research efforts have been directed toward ameliorating the inherently low electrical conductivity of cementitious systems, thereby enabling their utilization in smart applications such as self‐sensing,^[^ ^12^, ^13^, ^14^ ^]^ health monitoring,^[^ ^15^, ^16^ ^]^ information encryption,^[^ ^17^ ^]^ electromagnetic interference (EMI) shielding,^[^ ^18^, ^19^ ^]^ and self‐heating.^[^ ^20^, ^21^ ^]^ The pivotal role of conductive cement‐based materials in contemporary infrastructures, including roads, bridges, and buildings, has grown exponentially in recent years.^[^ ^22^, ^23^, ^24^ ^]^ The electrical performance of these materials is primarily influenced by the type of fillers and the formation of conductive networks.^[^ ^25^, ^26^, ^27^ ^]^

To realize high electrical conductivity in cement‐based composites, a substantial concentration of conductive fillers (carbon fibers (CFs), carbon nanotubes, graphene, and metallic constituents) is imperative, which surpasses the critical percolation threshold.^[^ ^25^, ^28^ ^]^ Nonetheless, once this percolation threshold is surpassed, an incremental enhancement in electrical conductivity through the addition of further conductive fillers becomes increasingly arduous.^[^ ^13^, ^20^ ^]^ Consequently, researchers have adopted a strategy of incorporating conductive fillers at varying scales (nano to micro) and morphologies (linear and flake) to form high‐conductive networks in cement‐based composites.^[^ ^20^, ^29^ ^]^ Despite the synergistic potential of combining various conductive fillers, the percolation threshold limits a further reduction in electrical resistance, and dispersion complexities within cement pastes act as a barrier to large‐scale applications.^[^ ^21^, ^30^ ^]^ Addressing these issues is crucial for advancing the development and practicality of high‐conductive cement‐based materials in the field of smart infrastructure.

Drawing inspiration from the popcorn effect, in which heat‐induced steam pressure causes significant volume expansion, we propose a novel stagey to enhance the electrical conductivity of conductive cement. Expandable graphite (EG) is produced by intercalating expandable agents between the layers of graphite crystals, allowing the layers to separate and expand, like popcorn, under high temperatures.^[^ ^31^, ^32^, ^33^ ^]^ The graphite expansion volume can promote the formation of new conductive pathways, thereby greatly reducing the overall resistance of the conductive cement. Notably, graphite still maintains excellent electrical conductivity after thermal expansion and exhibits excellent dispersibility in cement paste.^[^ ^34^, ^35^ ^]^ If EG is used as the conductive filler in conductive cement, electrical resistance can be reduced by reconstructing conductive networks even after reaching the permeation threshold, thereby enhancing the overall performance of conductive cement. This strategy has great potential for future cement‐based composites in terms of realizing the structure‐function integration and functional‐intelligence integration.

In this study, we introduce a novel strategy for the self‐propagating formation of EG‐based high‐conductive cement composites through a simple thermal treatment. The rapid expansion of graphite at high temperatures facilitates the contact between adjacent conductive fillers, causing the spontaneous formation of new conductive networks. This strategy, inspired by the popcorn effect method, has versatile applications in smart infrastructures. In this study, the chemical and physical evolution of conductive cement composites before and after heat treatment were systematically investigated. The mechanisms driving the reconstruction of conductive networks and the subsequent decrease in electrical resistance were elucidated. Compared with a standard EG‐based cement composite (GC‐25), a composite treated at 400 °C (GC‐400) exhibits a remarkable decrease in electrical resistance by ≈60% after heat treatment, broadening its scope in electrical applications such as joule‐heating, electrothermochromic applications, and EMI shielding.

## 2 Results and Discussion

### 2.1 Preparation and Structural Analysis

**Figure 1:**
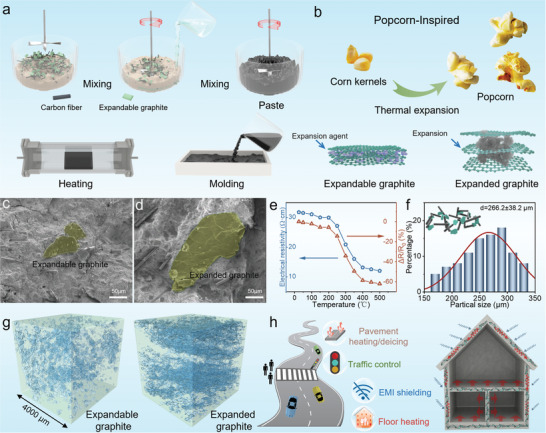
Fabrication and microstructure of cement composite. a) Schematic of the fabrication of graphite‐based cement composite (GC). b) Popcorn effect‐inspired thermal expansion strategies for graphite. c,d) Scanning electron microscopy (SEM) images of the cross sections of GC‐25 and GC‐400. e)Variation in resistance of the cement composite ranges from 20–500 °C. f) Graphite particle size before and after thermal expansion. g) 3D reconstruction models of graphite in GC‐25 and GC‐400 were built based on computed tomography (CT) images. h) Potential application concepts for smart cement composites.

**Figure** **1**a schematically depicts the preparation process of the expandable graphite‐based cement composite. This method leverages the rapid expansion of graphite at high temperatures, promoting contact between conductive fillers and forming new conductive networks (Figure 1b and S1, Supporting Information). The microstructures of GC‐25 and GC‐400 are shown in Figure 1c,d. The EG incorporated into cement composites showed volumetric variations before and after the heat treatment. As shown in Figure 1c, although the conductive fillers (i.e., CFs and EG) were dispersed uniformly within the cement matrix, some conductive fillers remained separated. This led to the formation of regions devoid of the microscale electrically conductive network, thereby affecting the electrical conductivity of GC‐25. Interestingly, the volume of the graphite increased after heat treatment (Figure 1d, Figures S2,S3, Supporting Information). When GC‐25 underwent heat treatment within the range of 25–500 °C, the variation in resistance of the cement composite was depicted in Figure 1e. The cement composite exhibited a low resistance change in the low‐temperature interval, this may be due to the deformation of graphite has not been fully triggered. As the temperature was further increased to 400 °C, the expandable graphite rapidly expanded and connected with adjacent carbon fibers, forming new conductive networks, which led to a sharp decrease in the resistance of the cement composite. However, since the graphite had essentially completed its expansion ≈400 °C, leading to the completion of the conductive network reconstruction in the cement composite, the resistance did not change significantly when the temperature was raised to 500 °C. Furthermore, excessively high heat treatment temperatures would lead to additional energy consumption and the deterioration of the mechanical properties of the cement composite. Therefore, heat treatment at 400 °C is the ideal temperature for preparing high‐conductivity cement composite. When the composite was exposed to high‐temperature environments, the average particle size of graphite expanded rapidly from 34 to 226 µm and facilitated contact between adjacent conductive fillers, leading to the self‐propagating formation of the composite conductive networks (Figure 1f and Figure S4, Supporting Information). A 3D reconstruction technology based on computed tomography (CT) images was used to rebuild the 3D model of graphite distribution and volume before and after heat treatment (Figures S5,S6, Supporting Information). As shown in Figure 1g, the 3D reconstruction models offer a highly intuitive visual impression of the graphite volume expansion from GC‐25 to GC‐400 (Videos S1, S2, Supporting Information). These newly formed conductive networks considerably improve the conductivity of the cement composite and broaden their applications in smart roads and buildings, such as pavement heating, floor heating, traffic control, and EMI shielding (Figure 1h).

### 2.2 Component Analysis and Physical Performance of GCs

**Figure 2:**
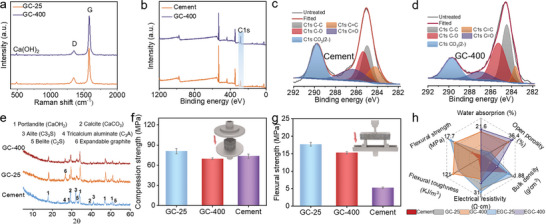
Physical properties and chemical compositions of GCs. a) Raman spectra of GC‐25 and GC‐400. b) X‐ray photoelectron spectroscopy (XPS) survey spectra of GC‐400 and pristine cement. (c,d) C1s XPS analysis of pristine cement and GC‐400. e) X‐ray diffraction (XRD) patterns of GC‐25, GC‐400, and pristine cement. f,g) Compression strength and Flexural strength of pristine cement and GCs. h) Comparison of the bulk density, open porosity, water absorption, electrical resistivity, flexural strength, and flexural toughness among pristine cement, GC‐25, GC‐400, EGC‐25, and EGC‐400.

The Raman spectra of GC‐25 and GC‐400 showed distinct characteristic peaks of portlandite (Ca(OH)_2_) induced by the cement matrix at ≈485 cm^−1^.^[^ ^36^ ^]^ Notably, two prominent peaks observed at ≈1350 and 1578 cm^−1^ are attributed to the presence of disordered carbon and ordered graphite crystalline structures, respectively (**Figure** **2**a).^[^ ^37^, ^38^ ^]^ This result confirms that conductive carbonaceous fillers (i.e., CF and graphite) are successfully incorporated into composites. In addition, X‐ray photoelectron spectroscopy (XPS) analysis was performed to investigate the carbon content and status of pristine cement and GC‐400. As shown in Figure 2b, compared with pristine cement, GC‐400 exhibited the C1s characteristic peak with a higher intensity at ≈284 eV because of incorporated CF and graphite.^[^ ^39^, ^40^ ^]^ The fitted C1s spectra of pristine cement and GC‐400 are shown in Figure 2c,d, respectively. Pristine cement had a higher ratio of CO_3_^2−^ than GC‐400, which is attributed to the presence of calcium carbonate (CaCO_3_).^[^ ^41^, ^42^ ^]^ With the introduction of CF and EG, the ratio of CO_3_^2−^decreased wheras the C─C bond increased. GC‐400 demonstrated a higher C─C bond ratio, proving that CF and graphite mixed satisfactorily in the cement composite.

The X‐ray diffraction (XRD) patterns of pristine cement, GC‐25, and GC‐400 cured for 28 days were obtained. The results indicate the existence of portlandite (Ca(OH)_2_), calcite (CaCO_3_), alite (C_3_S), belite (C_2_S), and tricalcium aluminate (C_3_A) in them (Figure 2e).^[^ ^43^, ^44^ ^]^ Because of the addition of carbon materials, the absorption peaks (2*θ* = 26.57°) of expanded graphite appeared in the XRD curves of GC‐25 and GC‐400.^[^ ^45^, ^46^ ^]^ The addition of carbon materials and heat treatment at 400 °C did not change the primary mineral composition of the cement matrix; however, after heat treatment, thermal expansion destroyed a part of the graphite structure primarily because of the gasification of interlayered insertion reagents. Thus, the characteristic diffraction peak associated with expanded graphite exhibited decreased intensity in the case of GC‐400 compared with GC‐25. As shown in Figure 2f, compared to the control of the pristine cement sample, the compression strength of GC‐25 slightly increased. This enhancement of compression strength can be mainly attributed to the crack resistance property and reinforcement effect brought by the addition of conductive fillers. This improvement in the intactness of GC composite can effectively mitigate stress concentration, thus making the stress load more uniformly distributed within the GCs when external compression is applied. It is worth mentioning that a slight decrease in the compression strength occurred when comparing the GC‐400 to GC‐25. One possible reason is that microcracks occurred in the cement matrix during the conversion of GC‐25 into GC‐400 triggered by heat treatment. This slightly intensified the uneven stress distribution when external stress was applied. Figure 2g shows the flexural strength of pristine cement and cement composites after curing for 14 days. The incorporation of CFs significantly improved the flexural strength and deformation resistance of GC‐25 and GC‐400 since the conductive fillers scatted inside reinforced the entire GC structure. It is notable that the flexural strength of GC‐400 also slightly decreased when compared to GC‐25. This decline in flexural strength can be also attributed to the fact that microcracks which can intensify local stress concentration were formed in the cement matrix due to the water evaporation during the heat treatment. Figure 2h shows a comparative analysis of six key performance metrics—flexural strength, flexural toughness, electrical resistivity, bulk density, open porosity, and water absorption. The presence of microcracks during the heat treatment of GC‐400, unlike the case in GC‐25, is correlated with increased water absorption, higher porosity, and reduced bulk density. Compared with pristine cement, the integration of CF and graphite endows GC‐25 with remarkable flexural strength, reaching 18 MPa, and enhanced flexural toughness. This implies that these composites can withstand substantial bending stresses and have a high capacity for energy absorption, which, in turn, leads to increased resilience against fracturing (Figures S7,S8, Supporting Information). The heat treatment process evidently induced a minor reduction in flexural strength in the GC‐400 composites. This slight weakening is attributed to the formation of microcracks that form as a byproduct of thermal processing. Regardless, GC‐400 maintained a flexural strength that significantly exceeds that of standard cement, highlighting its high conductivity and robust mechanical behavior. Moreover, GC‐400 had remarkable electrical conductivity compared to the samples without heat treatment, demonstrating their enhanced ability to conduct electricity. Because of the superior electrical conductivity coupled with dependable mechanical performance, the composites can serve as an innovative material in smart construction applications.

### 2.3 Electrothermal Performance of GCs

**Figure 3:**
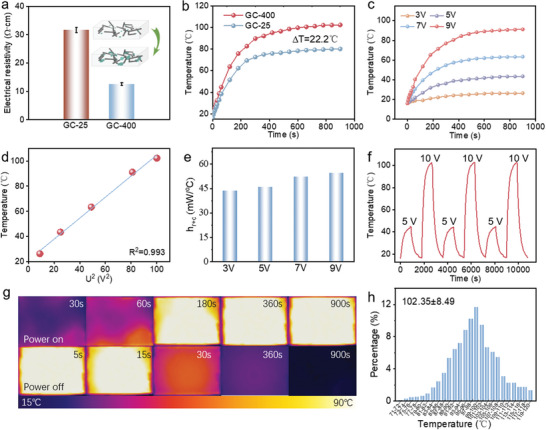
Electrothermal performance of GCs. a) Resistance of GCs. b) Average surface temperature of GCs at 10 V. c) Average surface temperature evolution of GC‐400 at different voltages. d) Relationship between the temperature and square of the applied voltage (*U* ^2^) relationship of GC‐400. e) Energy consumption of GC‐400 under different voltages. f) Cyclic performance of GC‐400. g) Infrared (IR) photographs and h) surface temperature distribution of GC‐400 at 10 V.

Restructuring of the conductive network induced by the self‐propagating formation of graphite imparts GC‐400 with excellent electrical conductivity. Compared to GC‐25, the electrical resistance of GC‐400 is ≈60% lower (≈12.6 Ω·cm). As shown in **Figure** **3**a and Table S1 (Supporting Information), the obtained cement composite exhibited high conductivity and reproducibility, with minimal resistance variability observed in the heat‐treated specimens (GC‐400). One possible reason for the resistance decrease is that the expanded graphite flakes can act as a bridge to connect the chopped carbon fibers in the vicinity, thus forming a directly connected conductive path and improving the conductivity of the network. In the scenario that neither the chopped carbon fibers nor the expanded graphite are in direct contact with each other, the expansion of expandable graphite can also lower the resistance via the enhanced “tunneling conduction” due to the decrease in the distances among the fillers.^[^ ^15^, ^26^, ^47^ ^]^ A lower resistance can make the cement composite more efficient in electrical‐thermal conversion. The average surface temperature of GC‐400 (≈100 °C) was 22.2 °C higher than that of GC‐25 under 10 V, as shown in Figure 3b. The average surface temperature evolution of GC‐400 for a complete heating cycle under different voltages was systematically evaluated (Figure 3c). Evidently, GC‐400 exhibited a typical joule‐heating performance, and the heating rate and stabilized average surface temperature increased with increasing applied voltages. GC‐400 also exhibited an excellent electrothermal performance, reaching ≈100 °C under a 10 V power supply. GC‐400 demonstrated an excellent electrothermal performance, which could achieve higher surface temperature with lower applied voltage, significantly exceeding that of previously reported cement composites (Figure S9 and Table S2, Supporting Information). Furthermore, a strong linear correlation (*R^2^* = 0.993) was observed between the stabilized average surface temperature and the square of the applied voltage (*U* ^2^), suggesting that the surface temperature was effectively controlled by adjusting the applied voltage (Figure 3d). For GC400, heat transferred through radiation and convection (*H_r+c_*) was positively correlated with energy consumption and was calculated using the average surface temperature evolution curve. The results revealed that GC‐400 had a low energy consumption, ranging 45–55 mW °C^−1^ at 3–9 V (Figure 3e). Heating–cooling cycle tests were conducted at 5 and 10 V to evaluate the heating stability and durability of GC‐400, as shown in Figure 3f. Throughout the test, the heating rate and stabilized average surface temperature did not change greatly, indicating the excellent stability and durability of GC‐400 for long‐term applications. The heat distribution of GC‐400 at 10 V was recorded using infrared (IR) photographs, as shown in Figure 3g. Because of the uniform dispersion of conductive fillers in the composite, the surface of GC‐400 had a uniform thermal distribution. To further analyze the thermal distribution of the composite surface, the temperature of each pixel in the IR photographs was recorded and statistically analyzed, and an accurate temperature distribution interval diagram was plotted (Figure 3h). The surface temperature of the composite followed a normal distribution; the distributions of surface areas with temperatures ≈100 °C were similar, proving that GC‐400 had a uniform heat distribution. Owing to its excellent electrothermal performance, durability, and uniform heat distribution, GC‐400 has high application potential for purposes such as floor heating, de‐icing, and indoor climate control.

### 2.4 Electrothermochromic Performance of EGC

**Figure 4:**
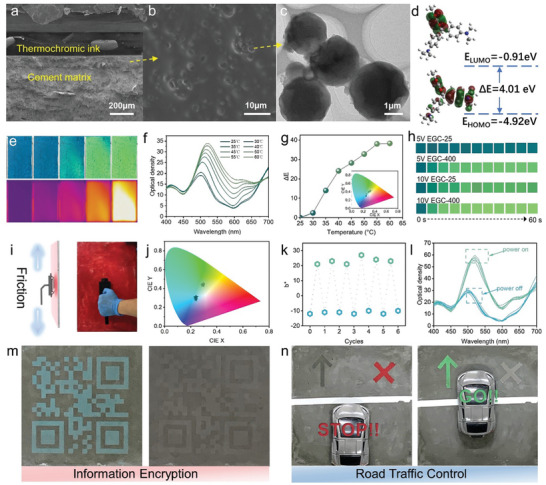
Electrothermochromic performance of EGCs. SEM images of a) the electrothermochromic ink layer deposited on the cement matrix and b) the enlarged surface of the electrothermochromic ink layer. c) Transmission electron microscopy (TEM) image of the electrothermochromic dye encapsulated in microcapsules. d) The least unoccupied molecular orbital (LUMO) and highest occupied molecular orbital (HOMO) of the major component in the electrothermochromic dye. e) The optical image and image of EGC. f) Optical images and the corresponding IR images of EGC samples captured at different heating temperatures, along with the responsiveness of the EGC sample color to heating. g) Optical density spectra of the EGC sample and h) shift in the color difference between the original color of EGC and its color at different temperatures. i) Schematic of the fastness of paint despite scrubbing. j) Reversible changes in the value of b* during repeated self‐heating. k,l) Spectra of the optical intensity and chromatic diagram of the paint during self‐heating. m) Information encryption enabled by the reversible change in the color of the QR code painted on the EGC sample n) the concept of traffic control by combined changes in the colors of the “arrow” and “cross” patterns.

As shown in the cross‐sectional SEM image (**Figure** **4**a), an electrothermochromic ink and putty formed a solid layer that was firmly anchored to the cement composite surface without any cracks or cavities (Figure S10, Supporting Information). Figure 4b shows microcapsules scattered uniformly inside the top layer of the electrothermochromic ink. As shown in Figure 4c, the microcapsules have an average size of 3 µm, and the mapping results confirm the existence of Si and O in the microcapsule shell and C and N from crystal violet lactone in the shell (Figures S11,S12, Supporting Information). Density functional theory calculation results are shown in Figure 4d; these results reveal that in crystal violet lactone, which is the primary component of the electrothermochromic ink, the highest occupied molecular orbital (HOMO) was localized on the phenyl unit, while the lowest unoccupied molecular orbital (LUMO) was localized on the lactone unit. The energy gap of the thermochromic ink was 4.01 eV, which can be easily driven by the conductive cement composite. As shown in Figure 4e, the color of EGC exhibits a typical fading effect, with its shade shifting from dark green to light green when it was self‐heated from room temperature 25 to 60 °C. This color change was manifested as the decline in the peak wavelength density during the self‐heating process (Figure 4f). The color difference (Δ*E*) between the original shade and shade captured at 60 °C reached as high as 38.25, demonstrating the wide color‐change range of the EGC sample (Figure 4g). Unlike the blue EGC sample, which exhibits a fading behavior during the self‐heating process, the red EGC sample underwent a typical color‐change process from red to yellow (Figures S13,S14, Supporting Information). This color‐change behavior was also evidenced by a shift in the wavelength of the maximum optical density (*λ_max_*) during the self‐heating process (Figure S15, Supporting Information). The color difference (Δ*E*) between the original shade and shade measured at 60 °C reached 70.16 (Figure S16, Supporting Information).

As shown in Figure 4h, a wider shade‐shift range was observed when the same voltage was applied to expanded samples than samples without any expansion. Upon electrical heating for 2 s, a remarkable change in the surface coloration of the EGC was observed, indicative of its fast response. This prominent change in the color of EGC can be mainly attributed to its distinct electrothermal ability, which triggered the electrothermochromic behavior of the paint. When the applied voltage was raised to 10 V, the shade of the EGC‐25 sample faded to light green. Furthermore, a lighter shade was observed when the same voltage was applied to the EGC‐400 sample. However, the final shade of the EGC‐400 sample when a voltage of 10 V was applied for 60 s was not much lighter than that of the EGC‐400 sample when 5 V was applied for the same duration. This indicates that the EGC‐400 sample exhibited a prominent electrothermal property, enabling the rapid color change of the electrothermochromic layer deposited on its surface.

The durability of the electrothermochromic layer coating on EGC was examined from the perspective of resistance to scrubbing and repeated heating. The electrothermochromic layer was soaked with water by wrapping the EGC board with a plastic film and sandwiching a folded wet cotton towel between them (Figure S17, Supporting Information). As shown in Figure 4i, a piece of black cloth was mounted on a wet‐scrub tester; the tester was then pushed against the wetted surface of the red EGC sample and rolled back and forth to repeatedly rub the black cloth against the painted coating. The painted electrothermochromic coating demonstrated satisfying fastness to crocking as no paint residuals or scratches were observed on the black cloth and red EGC sample surface (Figures S18,S19, Supporting Information). Furthermore, the coating exhibited stable color‐changing ability as the color measured at the same peak temperature remained almost the same when EGC was subjected to repeated self‐heating cycles (Figure 4j). This reversible color‐changing ability was evidenced by a cyclic shift in the b^*^ value in the range from −11.14 ± 0.34 to 23.17 ± 0.91 (Figure 4k and Figure S20, Supporting Information); furthermore, the cyclic maximum optical intensity changed from 29.12 ± 0.40 to 57.18 ± 0.59 (Figure 4l). A series of ECG‐400 specimens was obtained by painting various commercial thermochromic inks on its surface, and these specimens demonstrated a broad color gamut and various potential applications, and great stability (Figures S21–S25, Supporting Information).

A QR code was drawn by screen printing with the green electrothermochromic ink on the surface of the green EGC‐400 sample (Figure 4m). The QR code can be observed and scanned at room temperature by QR scanner apps installed on smartphones. When a voltage of 5 V was applied, the QR code was heated and turned to a lighter shade that could not be recognized by the QR scanner apps. This change in the shade of the QR code demonstrates the potential application of the EGC sample in information encryption. For traffic control applications, the combined symbols of “arrow” and “cross” can be printed on an EGC unit embedded in a road with two different electrothermochromic inks, and applying a certain voltage to the EGC unit triggers the synchronized color change of gray‐to‐green and red‐to‐light gray (Figure 4n). This color‐changing ability of the electrothermochromic EGC indicates its promising applications.

### 2.5 EMI‐Shielding Performance of CGs

**Figure 5:**
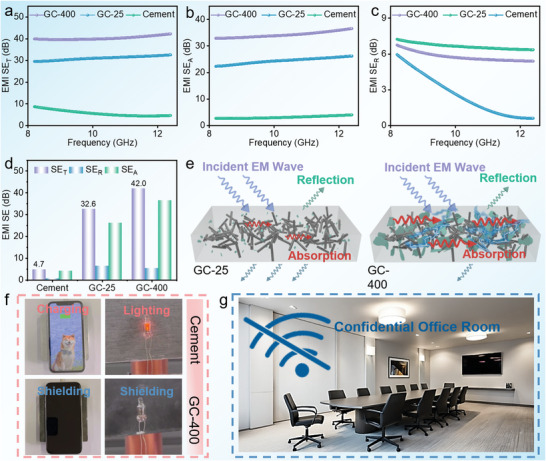
EMI‐shielding performances of GCs. a) EMI *SE_T_*, b) EMI *SE_a_*, c) EMI *SE_R_*, and d) EMI *SE*. e) Schematic of the EMI‐shielding mechanism of GC‐25 and GC‐400. f) EMI‐shielding application of GC‐400. g) Concept of a GC‐400‐based room for signal shielding.

The EMI‐shielding applications of traditional cement are highly restricted owing to their poor electrical conductivity. Pristine cement exhibited a low EMI‐shielding performance, yielding 4.7 dB in the range of 8.2–12.4 GHz, as shown in **Figure** **5**a. When CF and EG were added to the cement, the EMI‐shielding performance of the obtained composite (GC‐25) improved to ≈30 dB at 8.2–12.4 GHz (Figure 5b–d). This improvement in the electromagnetic shielding performance was mainly attributed to an enhancement in the absorbing shielding (*SE_A_*). However, the reconstruction of conductive networks due to the thermal expansion of graphite significantly reduced electrical resistance and further improved the EMI‐shielding performance of GC‐400. *SE_T_* reached ≈40 dB at 8.2 GHz and 42 dB at 12.4 GHz (Figure 5d). The mechanism underlying the enhancement in the EMI‐shielding performance for GC‐400 is shown in Figure 5e. When incident electromagnetic waves enter the cement composite, they are primarily absorbed through conductive loss by CF and graphite. Compared with GC‐25, the new conductive pathway formed in GC‐400 imparts better conductivity and a longer transmission path for electromagnetic waves, endowing GC400 with better EMI‐shielding performance. As shown in Table S3 (Supporting Information), GC‐400 exhibited excellent EMI‐shielding performance, essentially surpassing those reported for common building materials. Figure 5f intuitively shows the EMI‐shielding performances and potential applications of GC‐400. Because of the poor EMI‐shielding performances of pristine cement, the conductive coil can easily charge a mobile phone wirelessly (Video S3, Supporting Information). When GC‐400 was placed close to the coil, phone charging was interrupted immediately, demonstrating its EMI‐shielding performance (Video S4, Supporting Information). In addition, the same EMI‐shielding behavior was observed when the pristine cement and GC‐400 were placed between the coil and lamp (Videos S5,S6, Supporting Information). Therefore, the excellent EMI‐shielding performance of GC‐400 will enable the realization of the electromagnetic shielding of buildings, which has a wide range of application scenarios, including crucial meetings, interviews, examinations, and military applications (Figure 5g).

## 3 Conclusion

We developed a strategy for the self‐propagating formation of EG‐based high‐conductivity cement composites via simple thermal treatment inspired by the popcorn effect. We demonstrated the versatile applications of these composites in smart infrastructure. When EG was exposed to high‐temperature environments, the graphite expanded rapidly and facilitated contact between adjacent conductive fillers, resulting in self‐propagating formation of new conductive networks. Compared to standard EG‐based cement composite (GC‐25), these newly formed conductive networks in GC‐400 exhibited considerably lower electrical resistance (≈60% lower) after heat treatment, broadening the electrical applications of these composites. The as‐prepared GC‐400 exhibited considerably improved electrothermal performance, which was 22 °C higher than the average surface temperature of GC‐25 at 10 V. Furthermore, because of the excellent electrothermal performance and uniform heat distribution, GC‐400 can be used in potential electrothermochromic applications after anchoring with a solid electrothermochromic ink layer. A wider shade‐shift range with faster response (2 s) was applied to GC‐400, and potential applications such as information encryption and road traffic control were evaluated. In addition, GC‐400 showed excellent EMI‐shielding performance (42 dB at 12.4 GHz), essentially surpassing that of the pristine cement and GC‐25. The mechanism of performance enhancement was elucidated. This study paves the way for next‐generation buildings and infrastructures that are smarter, more functional, and better integrated with advanced electrical systems, setting a new benchmark for integrating modern technological advancements into the construction industry.

## 4 Experimental Section

### Materials

42.5‐grade ordinary Portland cement was procured from Huaxin Cement Company. The detailed chemical composition and physical characteristics of the cement were summarized in Table S4 (Supporting Information). CFs, with an average length of 3 mm, were dispersed within the EG graphite cementitious matrix as conductive fillers. The specific physical attributes of the CFs were summarized in Table S5 (Supporting Information). The EG, characterized by a D90 particle size of 200 mesh, was sourced from Qingdao Tengshengda Carbon Machinery Corporation, and further details are provided in Table S6 (Supporting Information). A silicone‐based defoamer, utilized as a defoaming agent, was acquired from China Dongyan Defoamer Corporation. Additionally, a polycarboxylate superplasticizer, specifically a modified polyether silicone (PCA‐I), was used as a water‐reducing admixture. It was obtained from China SBT New Materials Corporation. The electrothermochromic inks, with an average particle diameter of 1–10 µm, were supplied by Shenzhen Phantom Color Changing Technology.

### Preparation of Electrothermochromic Graphite‐Based Cement Composite

The procedure was as follows: 96 g of ordinary silicate cement and 3 g of EG were mixed in a planetary ball mill at a speed of 400 rpm for 4 h to obtain the mixture. Second, CF (1 g), water (a water‐to‐cement ratio of 0.2), a defoaming agent (0.2 mL), and a water‐reducing agent (1 g) were added into the mixed cement composite and stirred for 10 min at a speed of 300 rpm. Then, the obtained cement composite paste was poured into a rectangular polytetrafluoroethylene mold (30 × 50 × 4 mm) to fabricate the EG‐based cement composite (GC‐25). Due to the prices of EG and CF being only $77.22 and $25.28 per kg, respectively, this smart cement composite has a low cost. In each CG‐25 sample, the proportions of EG and CF were 3% and 1%, respectively, which only increased the cost by $0.0258 when compared to pure cement, with EG costing $0.023 and CF costing $0.0028. After the GC‐25 specimen was cured in a maintenance box (14 days, 25 °C, and 98% humidity), the specimen was dried in an oven at 60 °C for 24 h. Then, the specimen was heated in a muffle furnace (400 °C, 10 min) to ensure that the graphite expanded and reconstructed the conductive networks of the cement composite. The heat‐treated composite was renamed as GC‐400. In addition, the putty and diluted electrothermochromic ink solution were printed onto the front side of GCs to fabricate the electrothermochromic graphite–based cement composite. The obtained cement composites based on GC‐25 and GC‐400 were named as EGC‐25 and EGC‐400, respectively.

### Characterization

A scanning electron microscope (JSM‐7800, JEOL, Japan) was used to observe the microstructures and cross‐sections of the prepared composites. Micro‐CT (nanoVoxel‐2000, Sanying Precision Instruments Co., China) was used to analyze the volume of graphite before and after heat treatment. A universal testing machine (Instron 5967, Instron, USA) was employed to evaluate the flexural and compressive strength of the cured composites after a 14‐day curing period. The flexural strength was determined by testing the specimens in the same dimensions of 20 × 20 × 80 mm^3^ at a uniform loading rate of 0.02 mm min^−1^ with a support span of 60 mm. The compressive strength was evaluated by subjecting cubic specimens in the same dimensions of 20 × 20 × 20 mm^3^ to compression at a loading rate set at 1.2 mm min^−1^. For both flexural and compressive strength measurements, the results for the GCs were averaged across three individual samples. An XRD spectrometer (MiniFlex‐600, Rigaku, Japan) was used for phase composition and mineralogical examinations at a scanning speed of 10 per min. The phase compositions and carbon status of the composites were analyzed using a Raman spectrometer (InVia, Renishaw, UK). The chemical compositions of pristine cement and EGCs were estimated using an X‐ray photoelectron spectrometer (ESCALAB 250 Xi, Thermo Fisher, USA). The cement composites were coated with conductive silver adhesive at their extremities and clamped with steel clamps which were wired to the testing apparatus for both resistivity and electro‐thermal evaluations. The superior conductivity of the silver adhesive and steel clamps facilitated effective contact between the composites and the testing devices. A digital multimeter (34465A, Keysight, China) was employed to measure the electrical resistivity of the cement composites. To assess their electrothermal properties, the composites were powered by a direct current (DC) power supply (RXN‐305D, ZhaoXin, China). The heating tests were conducted at an ambient temperature of 25 °C. Surface temperatures during the electrothermal test were recorded and evaluated using IR photographs obtained with an IR camera (K20; HIKMICRO, China). The surface temperature distribution was investigated using the HIKMICRO Analyzer software. The color change in EGCs was evaluated using a spectrophotometer (CS‐650A, CHN Spec, China) under CIE standard illuminant D65. A vector network analyzer (N5244A, Agilent, USA) was used to measure the EMI‐shielding performance of GCs and pristine cement in the frequency range of 8.2–12.4 GHz.

## Conflict of Interest

The authors declare no conflict of interest.

## Supporting information



Supporting Information

Supplemental Movie 1

Supplemental Movie 2

Supplemental Movie 3

Supplemental Movie 4

Supplemental Movie 5

Supplemental Movie 6

## Data Availability

The data that support the findings of this study are available from the corresponding author upon reasonable request.
