# Supplementary material for: Popcorn Effect–inspired Self‐propagating Formation of High‐conductivity Cement Composite for Multifunctional Applications
Source: Adv Sci (Weinh). 2024 Dec 4;12(4):2411290. doi: 10.1002/advs.202411290 (PMC11775549; doi:10.1002/advs.202411290)
Supplement: Supplementary file 1 — Supporting Information [file ADVS-12-2411290-s005.docx]

**Supplementary Information**

**Popcorn Effect–inspired Self-propagating Formation of High-conductivity Cement Composite for Multifunctional Applications**

Haiping Wu, Jiaqi Huang, Zhengyao Qu, Xueling Zheng, Sirui Tan, Guanming Cai, Zhong Zhao*, Jing Wu* and Daiqi Li*

H. P. Wu, J. Q. Huang, X. L. Zheng, S. R. Tan, G. M. Cai, Z. Zhao, Prof. J. Wu and D. Q. Li

State Key Laboratory of New Textile Materials and Advanced Processing Technologies, Wuhan Textile University, Wuhan 430200, P. R. China.

E-mail: [zzhao@wtu.edu.cn](mailto:zzhao@wtu.edu.cn); wujing313@wtu.edu.cn; [dqli@wtu.edu.cn](mailto:dqli@wtu.edu.cn)

H. P. Wu, and Prof. Z. Y. Qu
State Key Laboratory of Silicate Materials for Architectures, Wuhan University of Technology, Wuhan 430200, P. R. China.

Figure S1 SEM images of expandable graphite and expanded graphite

Figure S2 Graphite distribution in the single-slice CT image of CG-25

Figure S3 Graphite distribution in the single-slice CT image of CG-400

Figure S4 Graphite particle size before thermal expansion

Figure S5 Graphite distribution in the stacked CT images of CG-25 captured at different view angles

Figure S6 Graphite distribution in the stacked CT images of CG-400 captured at different view angles

Figure S7 Compression strength–displacement curves of pristine cement and GCs

Figure S8 Flexural strength–displacement curves of pristine cement and GCs

Figure S9 Comparison of the electro-thermal performance of the GC-400 with those of reported building materials

Figure S10 Cross-sectional SEM image of the composite layer consisting of thermochromic capsules and putty

Figure S11 SEM image of the thermochromic microcapsules

Figure S12 Mapping results of the thermochromic microcapsule

Figure S13 Changes in the shade of red EGC sample from 25 ºC to 60 ºC

Figure S14 Chromaticity diagram of the shades of red EGC sample from 25 ºC to 60 ºC

Figure S15 Optical density spectra of the EGC red sample recorded from 25 ºC to 60 ºC

Figure S16 Color differences (ΔE) between the original shade of the red EGC recorded at 25 ºC and that recorded at different temperatures

Figure S17 Soaking the thermochromic layer with a folded wet cotton towel

Figure S18 Black cloth after the on-site wet-scrub resistance test

Figure S19 EGC-red sample before after scrubbing

Figure S20 Spectra of the optical intensity and chromatic diagram of the paint during self-heating with standard error bars

Figure S21 Optical images of the EGC samples pained with series thermochromic inks during the heating and cooling cycles

Figure S22 Chromaticity diagram of the various shades of series EGC samples from 25 ºC to 60 ºC

Figure S23 Color change of thermochromic pattern painted on the ECG sample during its self-heating cycle

Figure S24 The CIELab parameters measured on the different sampling points

Figure S25 Optical image of black cloth before and after the scrubbing tests

Table S1 Resistance change of the cement composites before and after heat treatment

Table S2 Comparison of the electro-thermal performance of the GC-400 with those of reported building materials

Table S3 EMI shielding performance Comparison with related building materials

Table S4 Chemical compositions and physical properties of the cement

Table S5 Physical properties of the carbon fiber

Table S6 Physical properties of the expanded graphite

Supporting Movie 1 3D reconstruction of expandable graphite distribution and volume in GC-25.

Supporting Movie 2 3D reconstruction of expanded graphite distribution and volume in GC-400.

Supporting Movie 3 Insufficient EMI shielding ability of pristine cement to shield wireless charging of the phone.

Supporting Movie 4 EMI shielding performance of GC-400 to shield wireless charging of the phone.

Supporting Movie 5 Insufficient EMI shielding ability of pristine cement to shield wireless lighting of neon lamp.

Supporting Movie 6 EMI shielding performance of GC-400 to shield wireless lighting of neon lamp.


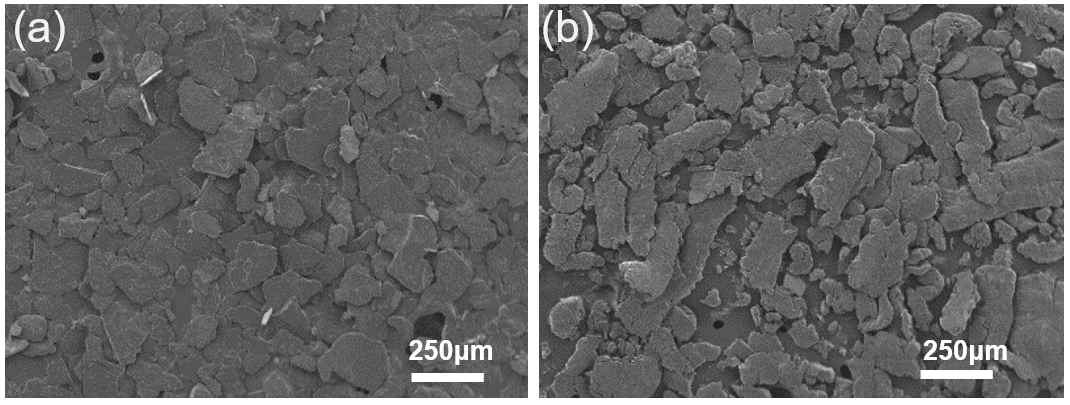


**Figure S1** SEM images of expandable graphite and expanded graphite


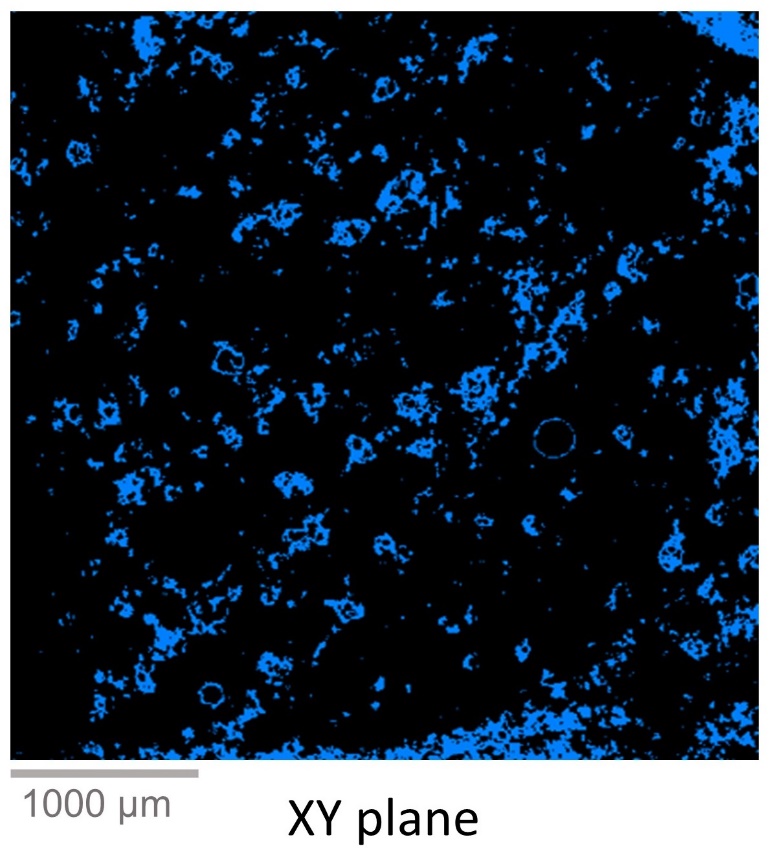


**Figure S2** Graphite distribution in the single-slice CT image of CG-25


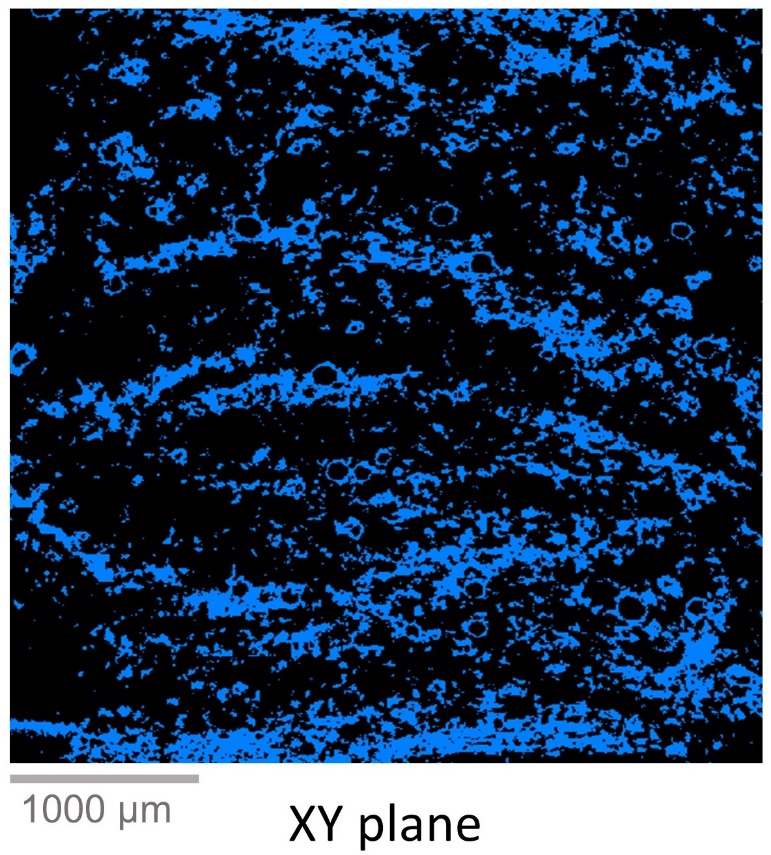


**Figure S3** Graphite distribution in the single-slice CT image of CG-400


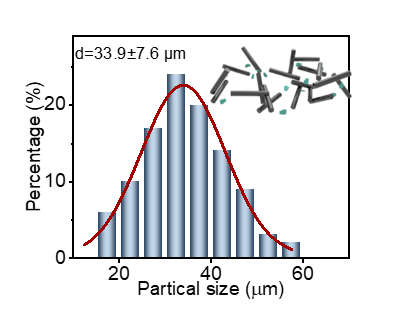


**Figure S4** Graphite particle size before thermal expansion


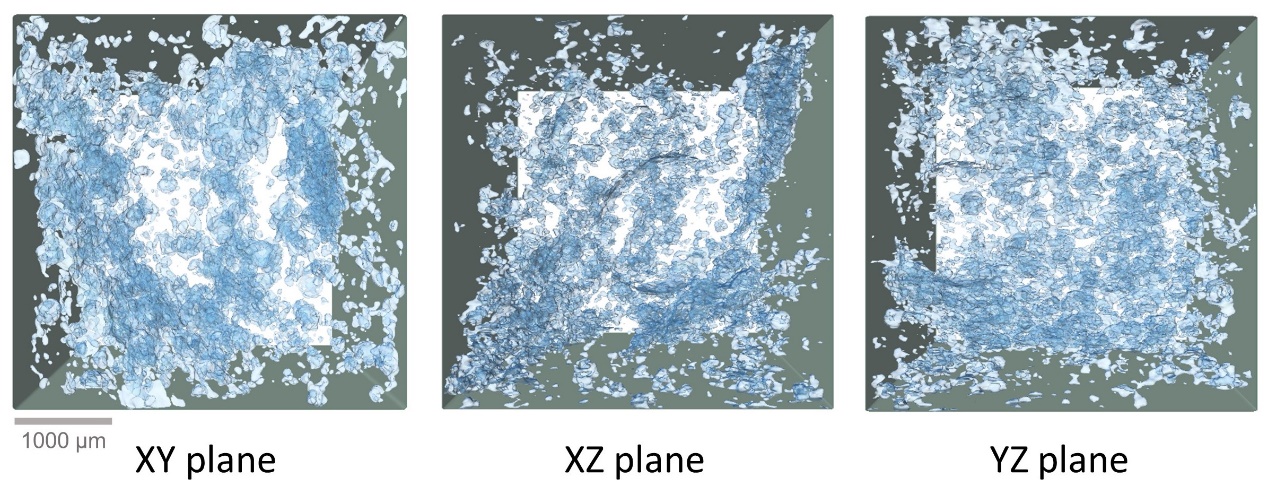


**Figure S5** Graphite distribution in the stacked CT images of CG-25 captured at different view angles


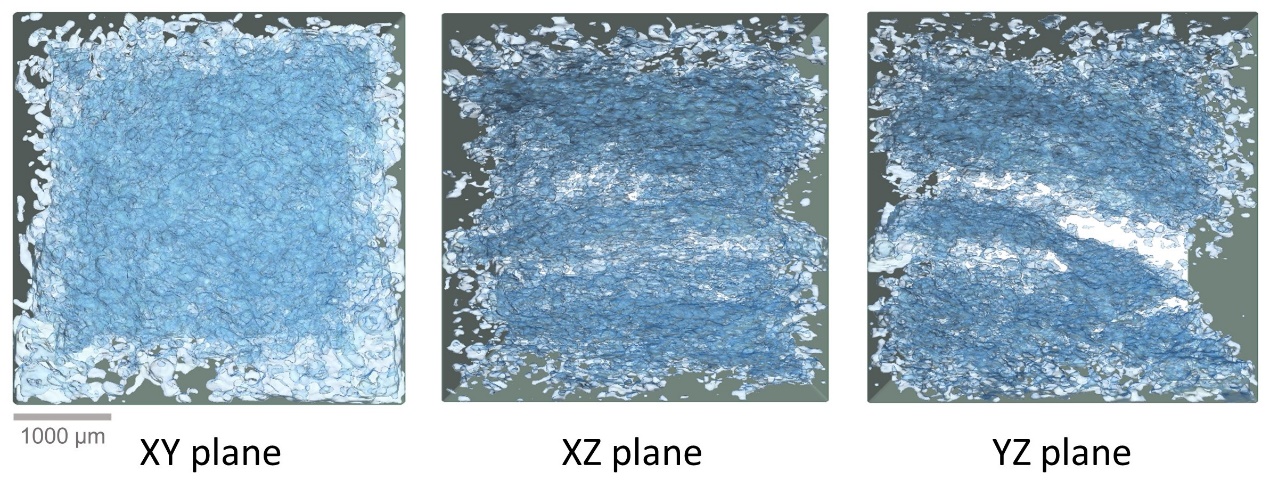


**Figure S6** Graphite distribution in the stacked CT images of CG-400 captured at different view angles


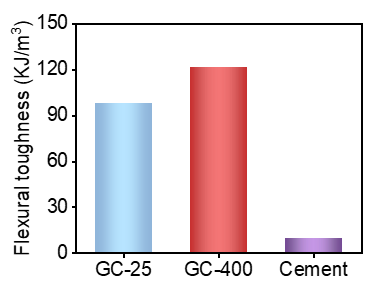


**Figure S7** Flexural toughness of pristine cement and GCs.


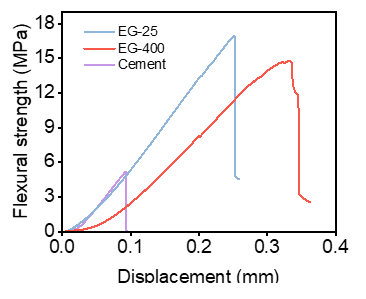


**Figure S8** Flexural strength–displacement curves of pristine cement and GCs.

**Figure S9** Comparison of the electro-thermal performance of the GC-400 with those of reported building materials.^[^^1-7]^


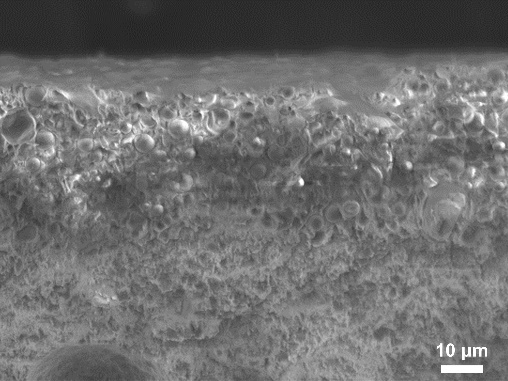


**Figure S10** Cross-sectional SEM image of the composite layer consisting of thermochromic capsules and putty


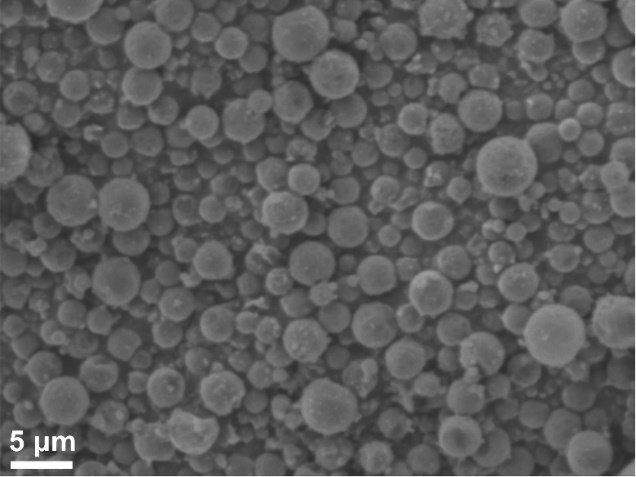


**Figure S11** SEM image of the thermochromic microcapsules


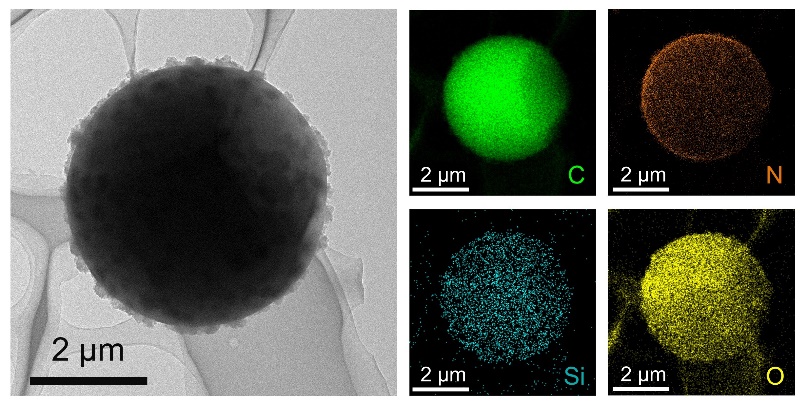


**Figure S12** Mapping results of the thermochromic microcapsule


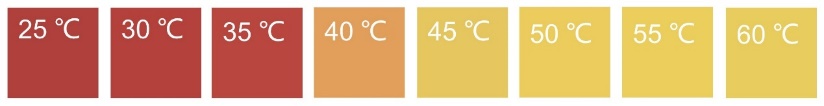


**Figure S13** Changes in the shade of red EGC sample from 25 ºC to 60 ºC


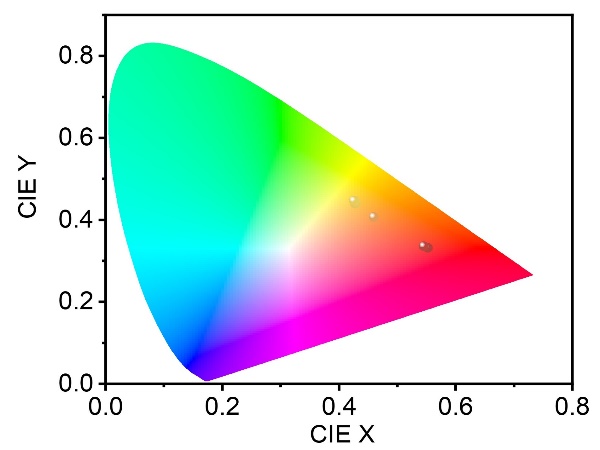


**Figure S14** Chromaticity diagram of the shades of red EGC sample from 25 ºC to 60 ºC


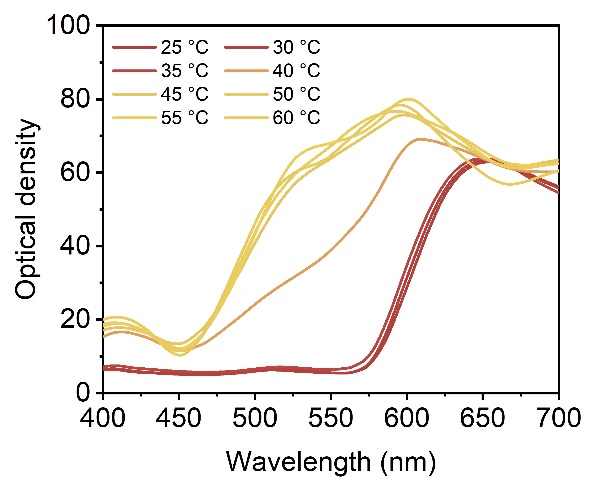


**Figure S15** Optical density spectra of the EGC red sample recorded from 25 ºC to 60 ºC


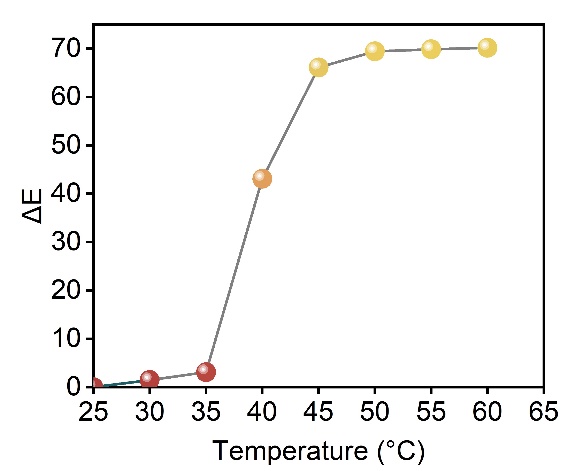


**Figure S16** Color differences (ΔE) between the original shade of the red EGC recorded at 25 ºC and that recorded at different temperatures


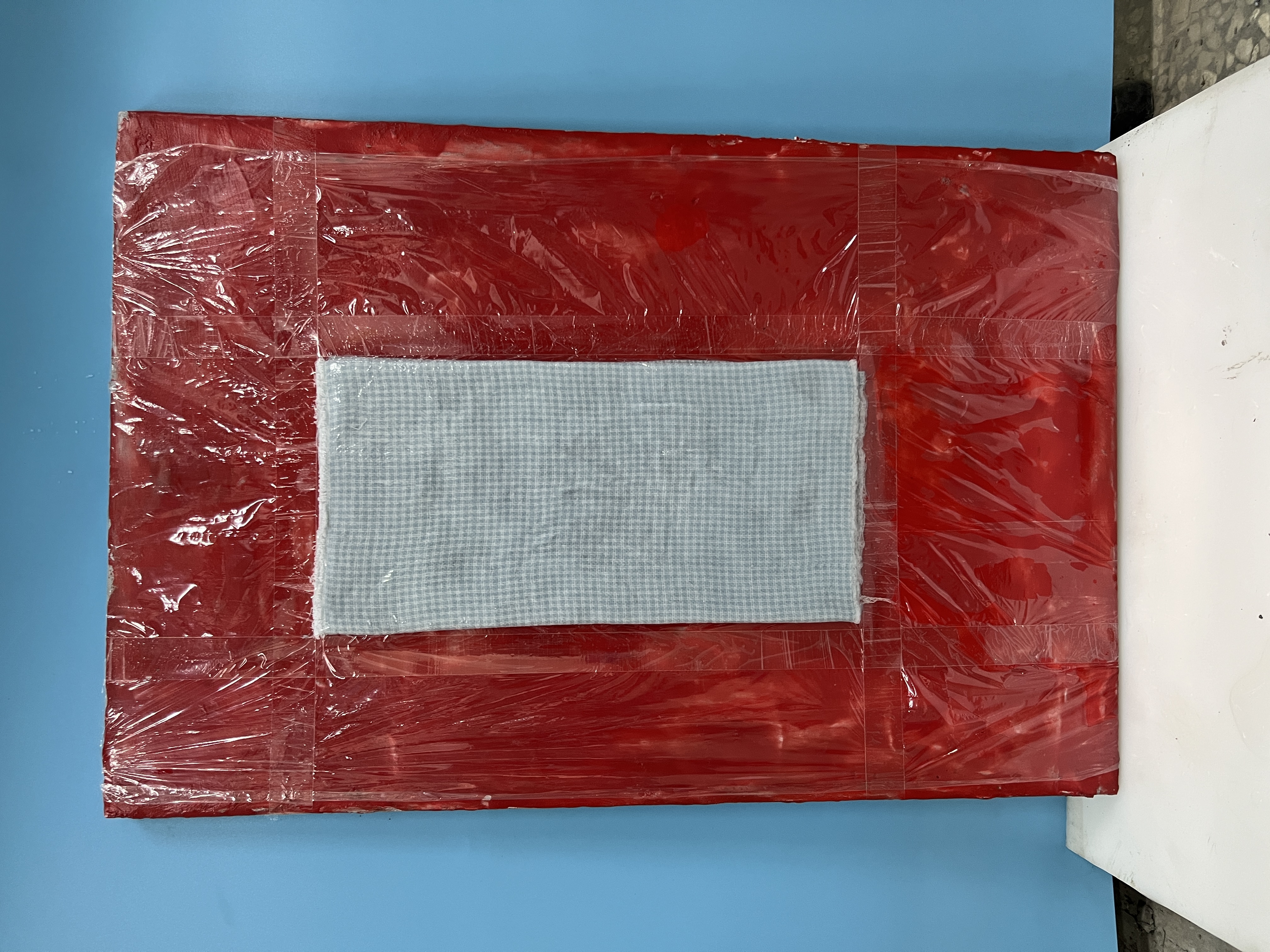


**Figure S17** Soaking the thermochromic layer with a folded wet cotton towel


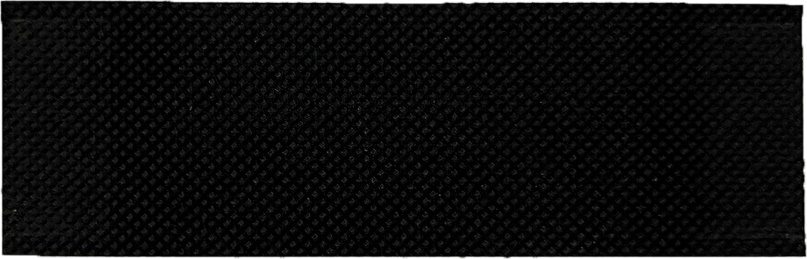


**Figure S18** Black cloth after the on-site wet-scrub resistance test


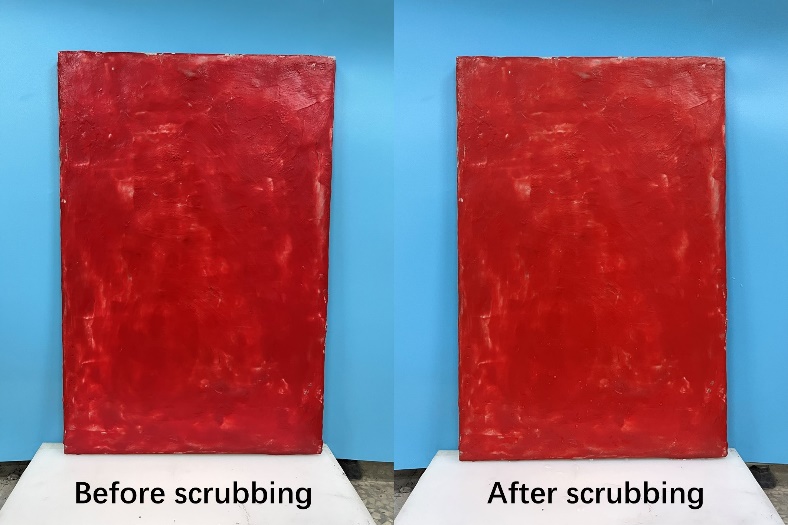


**Figure S19** EGC-red sample before after scrubbing


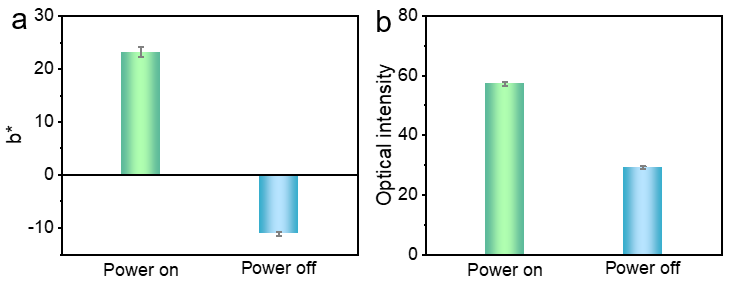


**Figure S20** Spectra of the optical intensity and chromatic diagram of the paint during self-heating with standard error bars


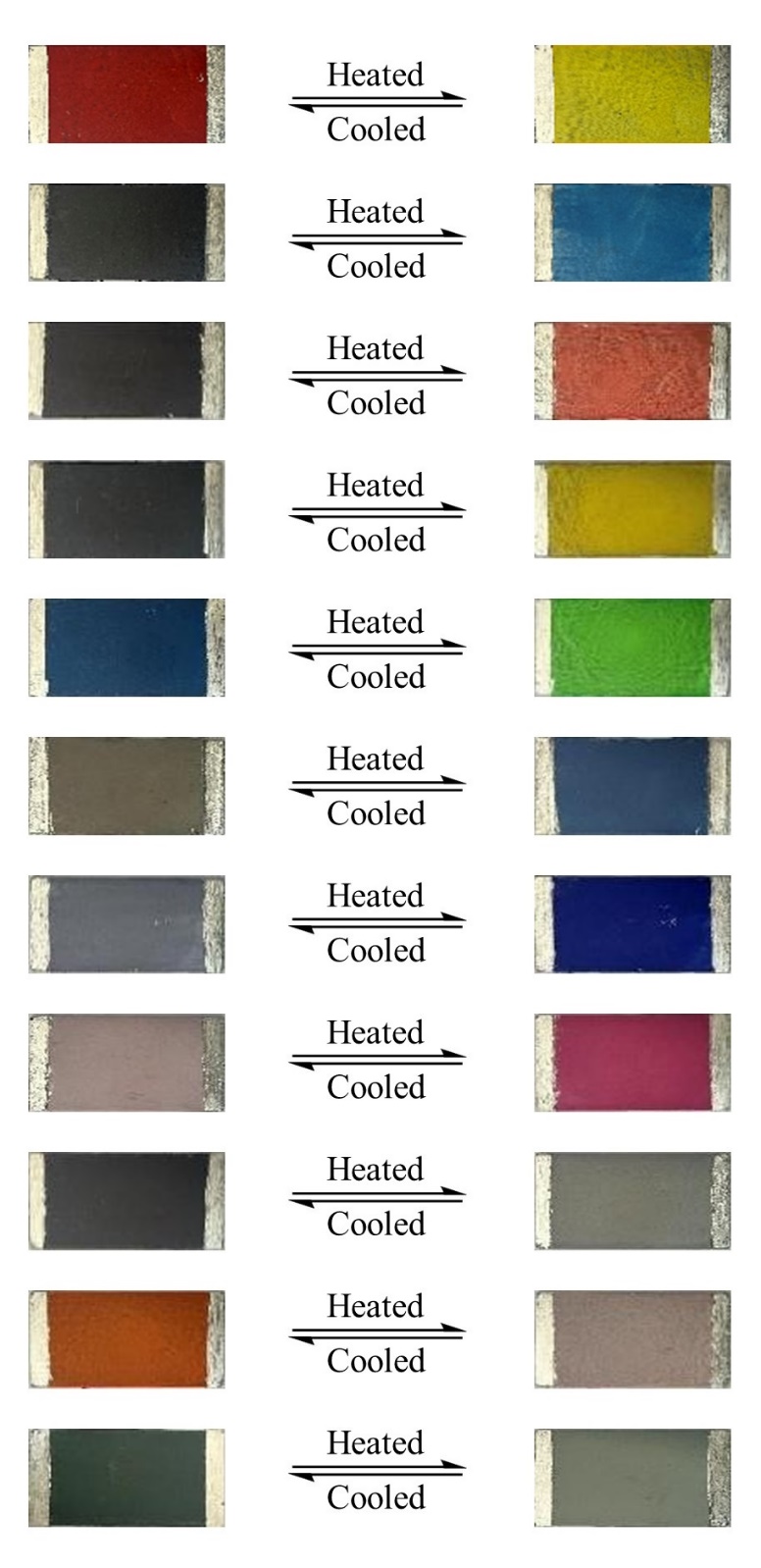


**Figure S21** Optical images of the EGC samples pained with series thermochromic inks during the heating and cooling cycles


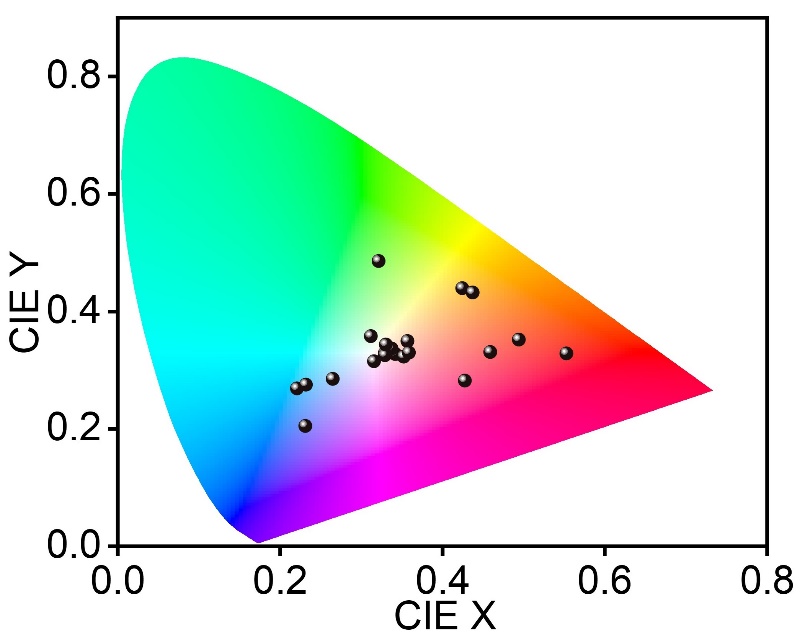


**Figure S22** Chromaticity diagram of the various shades of series EGC samples from 25 ºC to 60 ºC

**
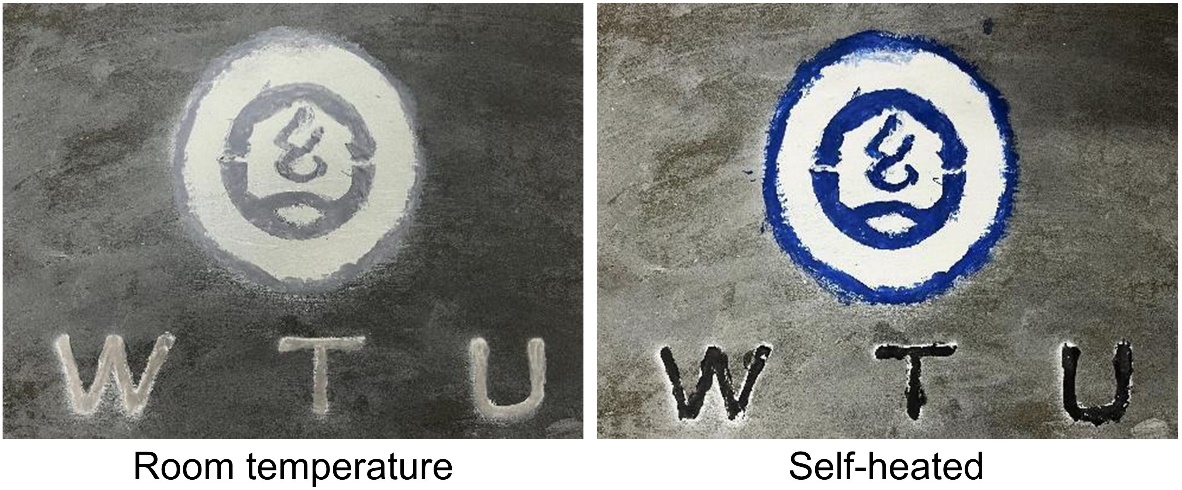
**

**Figure S23** Color change of thermochromic pattern painted on the ECG sample during its self-heating cycle


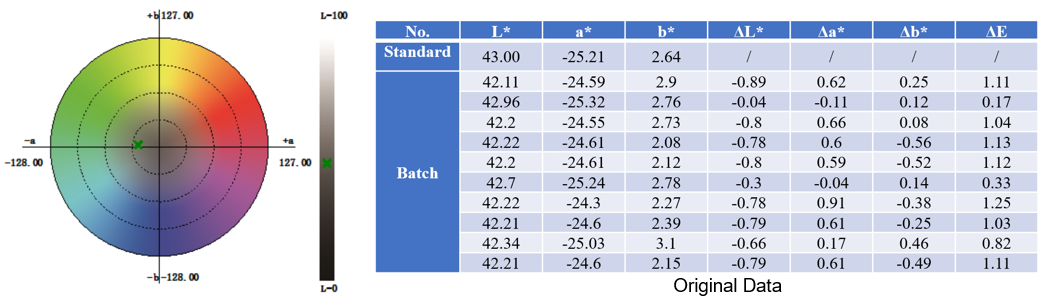


Figure S24 The CIELab parameters measured on the different sampling points and the original data

**
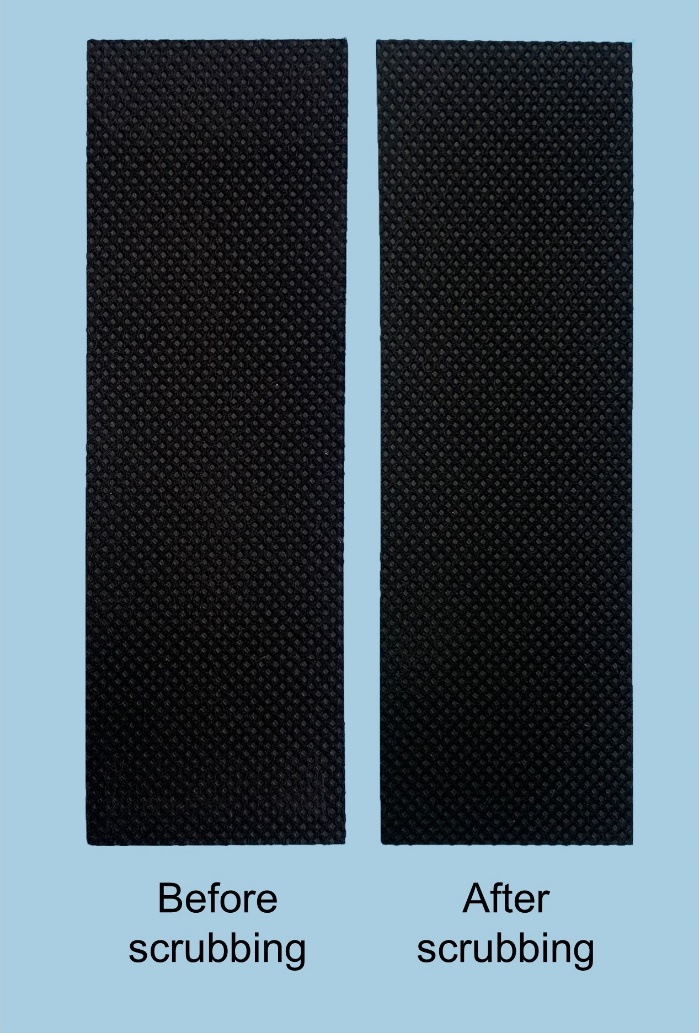
**

Figure S25 Optical image of black cloth before and after the scrubbing tests

**Table S1** Resistance change of the cement composites before and after heat treatment.

| **Sample ID** | **Electrical resistivity （Ω·cm）** | |
| --- | --- | --- |
|  | **GC-25** | **GC-400** |
| 1 | 34.6 | 15.5 |
| 2 | 28.8 | 10.6 |
| 3 | 32.5 | 13.2 |
| 4 | 31.9 | 12.4 |
| 5 | 27.8 | 10.8 |
| 6 | 32.3 | 13.3 |
| 7 | 36.2 | 12.7 |
| 8 | 29.8 | 12.6 |
| 9 | 33.7 | 13.2 |
| 10 | 28.8 | 11.2 |

**Table S2** Comparison of the electro-thermal performance of the GC-400 with those of reported building materials.^[1-7]^

| **Matrix** | **Filler** | **Content** | **Applied Voltage (V)** | **Temperature**  **(℃)** | **Ref** |
| --- | --- | --- | --- | --- | --- |
| **This work** | Carbon fiber/Expandable graphite | 3wt%EG＋1wt%CF | 10 | ~102℃ | / |
| Cement | Carbon fiber rope | / | 30 | ~100℃ | [1] |
| Cement Mortar | Carbon fiber | CF1.0wt% | 24 | ~45℃ | [2] |
| Cement Mortar | Carbon fiber | 2vol%CF | 12 | 77℃ | [3] |
| Cement Mortar | Carbon fiber | 2 vol % CF | 12 | 103℃ | [4] |
| Cement Mortar | Expanded graphite | 10wt%EG | 15 | 77.8℃ | [5] |
| Cement Mortar | Carbon fiber/carbon nanofibers | 0.75vol%CF＋0.3vol%CNF | 25 | 73℃ | [6] |
| Cement Mortar | Multi-walled carbon nanotubes | 0.5wt%MWCNT | 30 | 77℃ | [7] |

**Table S3** EMI shielding performance Comparison with related building materials^[8-14]^

| **Matrix** | **Filler** | **Content** | **Frequency** | **SE_T_ Value(dB)** | **Ref** |
| --- | --- | --- | --- | --- | --- |
| Cement | Carbonized tire | CWT-0.5% | 8-12 GHz | ~30 | [8] |
| Cement | CF/CNT | CF-0.3%/CNT-0.2% | 10 GHz | ~18 | [9] |
| Cement | Carbonized Bagasse fibers | PPN-0.5% | 8-12 GHz | ~20 | [10] |
| Cement | Copper | Copper - 0.25% | 8-12 GHz | ~16 | [11] |
| Ceramics | CF | CF-20% | 8-12 GHz | ~30 | [12] |
| Ceramics | Carbonized [phenolic resin](https://www.sciencedirect.com/topics/earth-and-planetary-sciences/phenolic-resin) | CCC-30% | 8-12 GHz | ~25 | [13] |
| Ceramics | Polycarbosilane | PCS-50% | 8-12GHz | ~37 | [14] |
| **This work** | Carbon fiber/Expandable graphite (GC-25) | 3%EG;1%CF | 8-12 GHz | **32.6** | **/** |
| **This work** | Carbon fiber/Expanded graphite (GC-400) | 3%EG;1%CF | 8-12 GHz | **42** | **/** |

**Table S4** Chemical compositions and physical properties of the cement

| Chemical compositions | Mass (wt%) | Physical properties |  |
| --- | --- | --- | --- |
| CaO | 63.01 | Starting set time | 213 minutes |
| SiO_2_ | 21.70 | Ending set time | 276 minutes |
| Al_2_O_3_ | 4.76 | Specific surface area | 3.01 g/cm^3^ |
| Fe_2_O_3_ | 3.57 | Blaine specific surface area | 359.9m^2^/kg |
| MgO | 2.00 | Compressive strength 3 day | 29.9MPa |
| SO_3_ | 2.49 | Compressive strength 28 day | 53.1MPa |
| Loss on ignition (LOI) | 3.91% |  |  |

**Table S5** Physical properties of the carbon fiber

| Diameter | Length | Carbon content | Density | Volume resistivity |
| --- | --- | --- | --- | --- |
| 7 μm | 3 mm | ≥95% | 1.78 g/cm^3^ | 2.0×10^-3^Ω/cm |

**Table S6** Physical properties of the expanded graphite

| Particle size (mesh) | Ash | Carbon content | | Expansion multiplier | pH value | Moisture |
| --- | --- | --- | --- | --- | --- | --- |
| 200 | 0.68% | 99.20% | 60% | | 5 | 0.07% |

**Reference**

[1]H. Wu, S. Tan, X. Zheng, Z. Zhao, M. Wang, Q. Ma, J. Wu, D. Li, *Materials &*

*Design* **2024**, 242, 113017.

[2]H. Dehghanpour, *Construction and Building Materials* **2023**, 406, 133449.

[3]Y. Liu, W. Tian, M. Wang, B. Qi, W. Wang, *Construction and Building Materials*

**2020**, 244, 118344.

[4]M. Hambach, H. Möller, T. Neumann, D. Volkmer, *Composites Part B:*

*Engineering* **2016**, 90, 465.

[5]M. Frąc, W. Pichor, P. Szołdra, *Journal of Composite Materials* **2020**, 54, 3821.

[6]W. Tian, Y. Liu, B. Qi, W. Wang, *Cement and Concrete Composites* **2021**, 117,

103904.

[7]H. Lee, W. Yu, K. J. Loh, W. Chung, *Construction and Building Materials* **2020**,

250, 118838.

[8]A. Mahmood, R. A. Khushnood, M. Zeeshan, *Journal of Cleaner Production* **2020**,

248, 119288.

[9]H. Yoon, D. Jang, H.-K. Lee, I. Nam, *Construction and Building Materials* **2021**,

269, 121238.

[10]A. Iftikhar, R. A. Khushnood, A. Khitab, S. Ahmad, *Construction and Building*

*Materials* **2023**, 370, 130722.

[11]Z. Yang, Y. Yao, Y. Zhuge, *Construction and Building Materials* **2024**, 426,

136162.

[12]Y.-M. Li, C. Deng, Z.-Y. Zhao, L.-X. Han, P. Lu, Y.-Z. Wang, *Composites Part A:*

*Applied Science and Manufacturing* **2020**, 131, 105769.

[13]D. Li, B. Tang, D. Cheng, J. Wu, W. Tang, Z. Zhao, J. Li, G. Cai, J. Wang, X.

Wang, *Engineering* **2023**, 21, 143.

[14]X. Liu, X. Yin, W. Duan, F. Ye, X. Li, *Journal of Materials Science & Technology*

**2019**, 35, 2832.
